# Recent advances on the role of host factors during non-poliovirus enteroviral infections

**DOI:** 10.1186/s12929-019-0540-y

**Published:** 2019-06-19

**Authors:** Collins Oduor Owino, Justin Jang Hann Chu

**Affiliations:** 1 Department of Microbiology and Immunology, National University of Singapore, Singapore, 117597 Singapore; 2 Institute of Molecular and Cell Biology, Agency for Science, Technology and Research (A*STAR), Singapore, 138673 Singapore

**Keywords:** Non-poliovirus enteroviruses, Hand, Foot and mouth disease, Human host factors, autophagy, Cell cycle arrest

## Abstract

Non-polio enteroviruses are emerging viruses known to cause outbreaks of polio-like infections in different parts of the world with several cases already reported in Asia Pacific, Europe and in United States of America. These outbreaks normally result in overstretching of health facilities as well as death in children under the age of five. Most of these infections are usually self-limiting except for the neurological complications associated with human enterovirus A 71 (EV-A71). The infection dynamics of these viruses have not been fully understood, with most inferences made from previous studies conducted with poliovirus.

Non-poliovirus enteroviral infections are responsible for major outbreaks of hand, foot and mouth disease (HFMD) often associated with neurological complications and severe respiratory diseases. The myriad of disease presentations observed so far in children calls for an urgent need to fully elucidate the replication processes of these viruses. There are concerted efforts from different research groups to fully map out the role of human host factors in the replication cycle of these viral infections. Understanding the interaction between viral proteins and human host factors will unravel important insights on the lifecycle of this groups of viruses.

This review provides the latest update on the interplay between human host factors/processes and non-polio enteroviruses (NPEV). We focus on the interactions involved in viral attachment, entry, internalization, uncoating, replication, virion assembly and eventual egress of the NPEV from the infected cells. We emphasize on the virus- human host interplay and highlight existing knowledge gaps that needs further studies. Understanding the NPEV-human host factors interactions will be key in the design and development of vaccines as well as antivirals against enteroviral infections. Dissecting the role of human host factors during NPEV infection cycle will provide a clear picture of how NPEVs usurp the human cellular processes to establish an efficient infection. This will be a boost to the drug and vaccine development against enteroviruses which will be key in control and eventual elimination of the viral infections.

## Introduction

Non-Polio enteroviruses belong to the genus *Enterovirus* (consisting of 15 species); family *Picornaviridae* [1] and have been identified in different parts of the world affecting human population [2]. Major outbreaks of non-polio virus associated infections have been recently reported in Asia Pacific, Europe, Canada and United States of America (USA). The peak of these infections is coming at a time when the world is nearing eradication of poliomyelitis, with just small number of cases reported in some parts of the world [3]. The burden of these infections has been felt in children under the age of five; most of whom are just beginning their early years at school. Most of these infections are known to be self-limiting but severe neurological complications and even death has been reported in some cases.

The focus of this review is to highlight the known role of human host factors and processes during the selected NPEV infections. A brief introduction on the epidemiology and pathogenesis of the selected non-polio viruses are described. The viral-host process/protein interactions are then discussed, followed by the existing gaps that need to be addressed in future. The ability of various NPEV viruses to usurp various cellular processes such as; cell cycle division, autophagy as well apoptosis, necroptosis and pyroptosis for efficient replication are also highlighted. The state of antiviral therapy research against these viruses is briefly discussed and existing gaps highlighted. The future perspectives and areas of concern are also emphasized.

### The burden of non-poliovirus enterovirus infections

Enterovirus A 71 (EV-A71) was first isolated from fecal and throat swab samples from patients with central nervous system complications in California [4]. Since then, EV-A71 has been linked with outbreaks of foot, hand and mouth disease (HFMD); often a self-limiting infection characterized with and severe forms characterized with acute flaccid paralysis and brainstem encephalomyelitis [5–8]. Coxsackievirus A16 (CV-A16), also plays a major role in hand, foot and mouth disease (HFMD) epidemics. Renal failure has also been reported in two HFMD cases due to CV-A16 infection [9, 10] and more recently one case of acute kidney injury secondary to EV-A71 infection was reported by Xu and colleagues [11]. HFMD outbreaks have been reported in different parts of Asia Pacific; often with neurological complications in children under the age of five especially in preschool centers as observed in Singapore [12]. For example, between 2008 to 2012 there were about 7.2 million probable cases of HFMD and about 2400 fatal cases reported in mainland China alone with high economic costs [13]. This year, 34 cases of encephalitis/neurological complications as a result of EV-A71 virus infection have been reported in Colorado, United States of America [14]. A 2–3 yearly cyclic pattern of hand, foot and mouth disease outbreaks have been reported in Asia pacific region [15]. The drivers of seasonality of NPEV in USA was studied recently by Pons-Salort and coworkers and identified the month of July and September to be the peak of these infections [16]. These outbreaks always result in the overburdening of the health care systems, pain and loss of lives in severe cases of the disease. Even though recent mathematical modelling findings using data from Singapore showed high incident rates with limited disability-adjusted life years (DALYs) compared to other infectious diseases prevalent in the South East Asian countries [17], HFMD has a potential threat to global health. Analysis of samples earlier collected for poliovirus surveillance studies in seven Western African countries identified several NPEVs circulating in the region with echoviruses being the dominant strain [18]. This study also identified least described types such as EV-A119, EV-B75, CV-A20 as well as EV-D94 among others to be circulating in this region [18]. The identification and molecular characterization of NPEVs in West Africa points to the global diversity of these viruses and calls for a stronger surveillance system for better management and control.

Recently, minor outbreaks of HFMD have been attributed to other coxsackieviruses such as CV-A10 and CV-A6. Even though the magnitude of their effects during outbreaks is not as large as that of EV-A71 and CV-A16; there is need to understand pathogenesis of the infections as well as quantifying their burden for easy disease monitoring. Coxsackievirus A6 (CV-A6) was isolated in USA in 1949 and has been recognized as one of the causative agents of hand, foot and mouth disease in different parts of the world including USA, Europe (Finland, Spain) and Asia Pacific (Taiwan, Japan, China, Thailand and Vietnam among other countries in the region) [19–27]. The emergence of CV-A6 as a player in the HFMD outbreaks eventually complicates vaccine and antiviral therapy development against HFMD. CV-A16 and EV-A71 have been broadly studied; however little success has been achieved in vaccine and therapy development thus the emergence of CV-A6 points to the urgent need of understanding its infection dynamics. Coxsackievirus A6 and A10 have been linked to sporadic outbreaks of atypical HFMD infections in China and France [28–32]. Between 2009 and 2011, CV-A10 and CV-A6 contributed to about 4.7 and 2.5% cases of HFMD correspondingly in China [32]. With time, CV-A6 has become one of the major causative agents of both severe and mild cases of hand, foot and mouth disease in China between 2013 to 2015; accounting for approximately 25.8% of mild and 16.9% of severe cases in 2015 [33]. There is a high possibility of virulent strains of HFMD viruses emerging as frequent recombination of enteroviruses A have been reported [30, 34]. These viruses have a potential of causing major outbreaks with potential threat to global health.

Enterovirus D 68 (EV-D68); first identified from throat swabs of children suffering from respiratory infections in 1962 and named as “Fermon virus” by Schieble and coworkers [35]. Since then, severe outbreaks of respiratory infections as a result of Enterovirus D 68 infections have been reported in Taiwan, USA, Canada and in Europe among other endemic regions [17, 36–38]. The link to acute flaccid paralysis and acute flaccid myelitis further exacerbates EV-D68 infections [39]. Several research studies have demonstrated the infection dynamics of this viral infection; for example, the ability of EV-D68 to infect neuronal cells has been reported by Brown and colleagues. Using neuronal cell line; SH-SY5Y confirming its neurotropism in line with the observed acute flaccid myelitis/ paralysis in patients [40]. Systemic and molecular diversity studies of EV-D68 in Lyon France, showed a diversification pattern for this virus [41]. The establishment of an experimental mouse model by Hixon and colleagues for studying the effects of EV-D68 provides much needed animal model for better understanding of the infection cycle of this virus [42]. Establishing the EV-D68 human host cell interactions will provide an insight into the pathogenesis of the infection and eventually be vital in the design of antivirals and vaccines against the virus.

There is necessity to extensively understand the molecular mechanisms of these viruses including the infection paradigms which will be key in the development of vaccines and antiviral therapy as well as players in molecular epidemiology.

### Host factors/processes involved in NPEV attachment, entry and internalizatione

Viral tissue tropism depends solely on cellular receptors which are responsible for attachment and entry of the virus particles into the host cells. Human host proteins act as receptors for viral attachment and eventual entry into the cells playing a role in the tissue tropism for various viral infections. Several receptors have been identified for various picornaviruses with poliovirus receptors being the first ones to be identified in this family. With the recent reemergence of enteroviral infection outbreaks, there is need to document all the recent findings in the entry process of these viruses; pointing to the eventual gaps that needs further research. Interplay between viral proteins and human host proteins play a major role in the attachment, entry and internalization of viral infections. Specific viruses use a confined set of receptors on the cell membrane for entry into susceptible cells, eventual uncoating of the virus. This process is vital for the eventual reproduction of viral genome and for continuity of the viral life cycle. Among the picornaviruses, poliovirus is the most extensively studied and several studies on non-polio enteroviruses have relied on these studies. A few host factors have been identified as possible receptors for the NPEVs, but the dynamics of the eventual attachment, entry and internalization is not yet fully understood.

Clathrin-mediated endocytosis as an entry pathway for EV-A71 virus was identified through siRNA screens targeting key genes involved in the process of endocytosis cytoskeletal dynamics, and endosomal trafficking [43, 44]. Since then it has always been known that clathrin mediated endocytosis is the major route of EV-A71 entry into susceptible cells. However, inhibition of the clathrin-mediated endocytosis pathways by chlorpromazine (CPZ) or dynasore (DNS) did not inhibit EV-A71 entry into the A549 cells, thus pointing to a combination of pathways involved in the viral entry [45].

Among the picornaviruses, poliovirus and rhinovirus receptors were identified in 1989; being the first enterovirus receptors to be described. Greve and his colleagues identified intercellular adhesion molecule 1 (ICAM-1) as a Rhinovirus receptor [46] while CD155 was described as a poliovirus receptor by Mendelshon and colleagues [47].

Some EV-A71 receptors have been identified; but these putative receptors have not been able to fully explain the diverse nature of symptoms observed in hand, foot and mouth disease cases. EV-A71 receptors include; human scavenger receptor class B member 2 (SCARB2); a known to not only function as an attachment receptor but also as an uncoating receptor during EV-A71 infection [48]. SCARB2 receptor is ubiquitously expressed in different parts of the body including neuronal cells. SCARB2 is a transmembrane receptor and a known β-glucocerebrosidase (β-GC) receptor responsible for transport from endoplasmic reticulum to lysosome and is also key in lysosome maintenance [49]. SCARB2 was also identified as an attachment receptor for human enterovirus species A and coxsackie A 16 virus [50].

Several cell types are known to express SCARB2, including the neurons thus may be directly linked to the neurological complications associated with EV-A71 infections; even though this has not been validated. At acidic and neutral conditions, the SCARB2 undergoes conformational changes leading to the opening up of lipid transfer channel mediating ejection of hydrophobic pocket from the virion a process important for viral uncoating [51].

P-selectin glycoprotein ligand-1 (PSGL1) a membrane protein expressed on white blood cells where it is responsible for inflammation, tethering or rolling of leukocytes at the vascular endothelial has also been described as a receptor for EV-A71 responsible for the viral entry into blood cells [52–54]. PSGL-1 has a high avidity for EV-A71 virus compared to SCARBR2 yet its associated with low infection effeciency due to its inability to induce viral uncoating [55].

Sialyated glycans were also elucidated to be playing a role in EV-A71 infection of the DLD intestinal cells [56]. Another attachment receptor; heparan sulfate glycosaminoglycan was also identified by Tan and colleagues pointing to the number of binding options available for EV-A71 virus [57]. A recent study by Tseligka and coworkers confirmed the importance of heparan sulfate during EV-A71 infection [58]. This explains the wide range of symptoms associated with EV-A71 infections from mild infections to neurological complications in some cases. Yang and colleagues identified the interaction between EV-A71 viral protein 1 (VP1) and human annexin 2 protein thereby enhancing EV-A71 infection [59]. Cell surface vimentin has also been described as attachment receptor for EV-A71 pointing to the presence of array of receptors responsible for the viral entry into the cells [60]. Using glycoproteomic approach, Su and colleagues identified cell surface nucleolin to be aiding in EV-A71 attachment and entry by interacting with viral protein 1 [61]. Cell surface prohibitin was recently identified as the first possible host factor that interacts with EV-A71 during viral entry into neuronal cells thereby aiding in the neuropathies associated with EV-A71 infections [62]. Fibronectin; a high molecular weight glycoprotein joins the list of the wide array of EV-A71 receptors to be discovered recently by Qiao and colleagues [63]. This study postulates that EV-A71 may be binding to the fibronectin protein through its VP1 structural protein.

A recent genome-wide RNAi screening by Yueng and colleagues identified human tryptophanyl-tRNA sythetase (hWARS) as an entry factor for EV-A71 as well as CV-A16 and EV-D68 [64]. The results from this study proposed an interesting view as the hWARS are not anchored on the membrane surface where it may be acting as a receptor; thus, there is need for further studies to unravel the exact mechanism of action of these proteins. As suggested by Perlman and Gallagher [65] in their commentary review on the findings from Yueng’s group, we support the need to further evaluate mechanisms of the three known EV-A71 entry receptors to find out if there is any interactions or if they are all needed for effective entry of the virus into susceptible cells. Possible mode of action for this new perspective in EV-A71 infection has been extensively reviewed in the commentary issue by Perlman and Gallagher [65]. Given that EV-D68 and CV-A16 viruses do not depend on PSGL1 and SCARB2 receptors for entry into cells, the findings of this study will be key in understanding the pathogenesis of these viruses upon validation of the exact mechanism of action. This was the first report linking interferon gamma to inducing viral entry into the cells.

The continued research aiming at documenting the array of receptors for EV-A71 and other picornaviruses will provide vital information in design of antiviral therapies and vaccines. Completely mapping out all the essential host proteins acting as functional receptors for EV-A71 will provide a rich niche for design and development of vaccines and therapy against infections associated with it. The existing EV-A71 and CV-A16 receptors have not been able to completely explain the pathogenesis of hand, foot and mouth disease. Human PSGL1 for example seems to only facilitate a small number of enteroviral entry into the cells, while SCARB2 has been shown to support an array of the viruses. This points out to the need of a more concerted efforts to identify and establish all the possible functional entry receptors for EV-A71. The recently identified hWARS needs to be further be validated to determine the efficiency in supporting entry of the enteroviruses reported from this study. Much need to be done going forward to fully understand the pathogenesis of the hand, foot and mouth disease. With a full map of entry receptors or factors, we will be able to design antiviral therapy able to block the entry pathway of the viruses thus limiting viral infections. This will be important in the design of antivirals against enteroviruses associated with the hand, foot and mouth disease.

Sialic acid as well as Intercellular adhesion molecule- 5 (ICAM 5) have been identified as receptors for enterovirus D68 (EV-D68) facilitating entry into susceptible cells [66, 67]. The coxsackievirus-adenovirus receptor (CAR) protein was the first receptor to be identified for coxsackie B virus subgroups A, C, D E and F [68, 69]. Thereafter, other receptors for Coxsackievirus A 24 and coxsackievirus A24 variant (CV-A24v) responsible for acute hemorrhagic conjunctivitis (AHC) have been described. ICAM-1 was identified as an uncoating receptor for CV-A21; sialic acid as an attachment receptor for CV-A24v [70]. Low density lipoprotein receptor (LDLR) was purified by Hofer and coworkers from HeLa cell culture supernatant and classified as minor rhinovirus receptor [71]. Very low lipoprotein receptor was also identified to be a receptor of the human rhinovirus 2(HRV2) [72]. Intercellular adhesion molecule-1 (ICAM-1) was also observed to be aiding infection of mouse cells by coxsackievirus A21 and rhinovirus thereby acting as its receptor [46, 73].

Another host factor; KREMEN1 was recently shown to play a role in the entry of coxsackievirus A10 (CV-A10); serotype A enterovirus [74]. This study also showed that KREMEN1 played a major role in entry of other serotype A enteroviruses; A2, A3, A4, A5, A6 and A12 [74]. Interestingly sequence analysis of these viruses using the enteroviral structural protein P1 showed that they cluster together on the phylogenetic tree.

Studies on another enterovirus; rhinovirus C (RV-C), associated with severe respiratory diseases, wheezing and asthmas in children has been limited by the inability to grow in cell cultures. However a recent study identified human clathrin related family member 3 (CDHR3) as a functional receptor for the RV-C [75]. Receptors for both the major group of rhinoviruses A and B have been described. Major group of rhinovirus A and B (RV-A and RV-B) binds to the intercellular adhesive molecule (ICAM-1) [46] while the minor group binds to the low density lipoprotein for efficient entry into the cells [71, 76].

Identification of receptors for the enteroviruses enables us to understand the pathogenicity of these epidemiologically important group of viruses. Attachment, adsorption and entry of viruses into the cells are the key initial stages for establishing efficient viral infections. There is need to understand the infection-mics of the rhinoviruses with a goal of developing antivirals or vaccines towards this group of viruses. For echoviruses; decay accelerating factor (DAF); CD55 known to regulate complement system within cells was also shown to be a receptor for a number of echoviruses and coxsackie B viruses [77–79]. Known NPEV receptors are summarized in Table 1 below.

**Table 1:** NPEV receptors

Receptor/entry factor	Viruses	Cell line/animal model used for the study	Reference
Decay accelerating factor (DAF)	Echovirus, Coxsackievirus B3	HeLa, T84, WOP	[77–79]
Intercellular adhesion molecule-1 (ICAM-1)	Coxsackievirus A21, rhinovirus, CV-A24	Mouse L cells, HeLa B, HAP1, HeLa R19	[46, 70, 73]
Sialylated glycan	EV-A71	DLD-1 intestinal cells, K562 myeloid cells	[56]
Annexin	EV-A71	HepG2	[59]
Heparan sulfate glycosaminoglycans	EV-A71, CV-A16	Chinese hamster ovary cells (CHO), rhabdomyosarcoma (RD)	[57]
Human scavenger receptor class B member 2	EV-A71, CV-A16, CV-A7, CV-A14	Mouse L929, Ltr929, Ltr245, Ltr051, RD	[48, 50]
Human P-selectin glycoprotein ligand 1	EV-A71	CHO-K1, RD, Jurkat, MOLT4, MT-2, HEp-2	[52, 53, 80]
Intercellular adhesion molecule-5	EV-D68	HEK293T	[66]
Vimentin	EV-A71	U251, RD, Vero, HeLa	[60]
Cell surface nucleolin	EV-A71	RD	[61]
Sialic acid	EV-D68,	RD, HAP1, human lung fibroblast (HELF)	[67]
LDLR, VLDLPR	Human Rhinovirus minor	HeLa	[71, 72]
Fibronectin	EV-A71	RD, HEK293	[63]
KREMEN1	CV-A10, CV-A2, CV-A3, CV-A4, CV-A5, CV-A6 and CV-A12	HAP1, H1-HeLa, HEK293, HCT 116, Vero E6, RD; *Kremen1/2*-Deficient Mice	[74]
hWARS	EV-A71, CV-A16 and EV-D68	human neuronal NT2, mouse fibroblast L929	[64]

Clearly dissecting the human host cell factors-NPEV interactions will provide a rich niche of interaction map that will be key in the design of antiviral therapy against this group of epidemiological importance. Understanding the mechanisms involved in viral entry as well as the host cell factors acting as receptors will provide important information on the development of viral entry inhibitors. Given that most of these viruses use an array of host factors/mechanisms to infect the host cell, as blocking of known entry inhibitors do not completely inhibit viral entry into the cells. This supports the need to clearly elucidate and map out all host factors involved in the viral attachment and eventual entry. This interaction between human host factors and viral proteins for eventual entry into the cells plays the key role in the viral tissue tropism. We therefore suggest that more concerted efforts need to be put in place to identify all possible entry mechanisms of these viruses with an aim of developing NPEV entry inhibitors into the cells thereby limiting viral infection. This can only be fruitful if we eventually identify all the host factors needed for the NPEV entry into cells.

### Host factors plays a role in viral NPEV virus genome replication

The recent technological advances have been essential in high throughput genome-wide screens aimed at discovery of the interplay between human host factors and the steps involved in viral infection. These techniques have revolutionized the identification of human host factors involved in viral infections with much success so far. Cherry and Panda presented techniques for siRNA genome-wide screens, detailing all the basic steps involved [81]. Several studies have used the siRNA genome-wide screens to identify the role of human host factors during enteroviral infections. Wu and colleagues performed a siRNA genome-wide screen which identified several human host factors necessary for EV-A71 virus infection [82]. This study identified susceptible host factors and resistant host factors involved in EV-A71 infection; NGLY1 and CDK6 and AURKB respectively pointing to an important interaction between viral proteins and human host cell factors.

A small siRNA screen targeting human membrane trafficking genes identified vasolin-containing protein (VCP-p97) as an important protein essential after PV viral replication and it interacts and colocalizes with 2 BC/2C as well as 3AB/3B in poliovirus infected cells [83]. EV-A71 through 2A^pro^ and 3C^pro^ have been shown to target endoplasmic reticulum proteins thereby leaving the ERAD proteins tethered within the ER lumen [84]. EV-A71 2A^pro^ specifically inhibits synthesis of Herp and VIMP at the translational level, while 3C^pro^ cleaves Ubc6e at Q219G, Q260S, and Q273G thereby interfering with the ERAD processes [84]. This study proposed that EV-A71 may be interfering with the ER membranes and hijacks ERAD component; p97 to improve its replication [84]. Pharmacological inhibition of myristoyltransferases resulted in a decreased myristoylation of CXB3 virus structural proteins through reduction of VP0 acylation [85]. Inhibition of myristolyation by siRNA knockdown and use of myristic acid analogues prevented cleavage between VP4 and VP2 as well as reduction in viral RNA synthesis [86]. These studies brings forth a new mechanism of myristoylation in picornaviral protein cleavage and processing of VP0 thus providing an alternative target for possible antivirals against these viruses [85].

RNA viruses have evolved with the human host cells to devise mechanisms of protecting themselves from the hostile environments within the host. These interactions result in the protection of the viral RNA integrity for an efficient infection and eventual establishment of disease as reviewed by Barr and Fearns [87]. It is a common belief that RNA viruses can remodel their host cell intracellular membranes to form double membranous structures; replication organelles that acts as a replication site for their genome. However, the mechanism of host cell remodeling has not been fully explicated. The sequential events leading to the formation of replication organelles are not yet fully identified. There is need to elucidate the role of the human host factors especially the lipid transfer proteins within the endoplasmic reticulum. It has been postulated that enteroviruses usurp the lipid transfer at the membrane to aid in the formation of the replication organelles [88]. Stoeck and his colleagues showed that Hepatitis C virus (HCV); positive stranded RNA virus usurps lipid transfer protein Neimann pick type C1(NPC1) within the late endosomes where it leads in localization of cholesterol leading to the formation of the double membrane structures essential for the formation of the replication organelle [89]. It will be important to elucidate the role of other known lipid transport proteins including steroidogenic acute regulatory protein (StAR) and Oxysterol-binding protein-related protein 1A and B (OSBPL1A) in the formation of replication organelle during NPEV viral infections.

Hsu and colleagues showed how viruses usurp host processes and proteins to reorganize host membranes to form replication organelles via the reorganization of the secretory pathways [90]. This study showed how enteroviruses and flaviviruses exploit host machinery; Arf1 and GBF1 resulting in recruitment of phosphatidylinositol-4-phosphate(PI4P) lipid augmented organelles vital for their replication [90]. Specifically, this study showed that enterovirus RNA polymerase binds PI4P thus illustrating the importance of phosphoinositide lipids during viral genome replication.

Zhang and colleagues elucidated that ARF1 and GBF1; vesicular proteins colocalizes with phosphatidylinositol-4-kinase IIIβ(PI4PIIIβ) leading to accumulation of PI4P thus pointing to their essential role during HCV virus infection [91]. This far, it has been shown that enteroviruses recruit PI4PIIIβ via the 3A viral protein for efficient viral genome replication. A study by Dorobantu and colleagues highlighted that the recruitment of PI4PIIIβ to the replication organelle does not depend on the interactions of GBF1/ARFA and acyl coenzyme A [acyl-CoA]-binding protein domain 3 (ACBD3) during coxsackievirus B3 replication [92]. Thus, the mechanisms of recruiting the PI4P leading to subsequent formation of replication complex remains unclear.

Furthermore, studies by Xiao and coworkers showed that EV-A71 3A protein facilitates the interaction between ACBD3 and PI4PIIIβ at the replication sites [93]. Contrary to previous studies showing that PI4PIIIβ recruitment is independent of ACBD3 during rhinovirus infection, this particular study points to a selective recruitment strategy of PI4PIIIβ facilitated by 3A protein to the replication sites during EV-A71 infections [93].

A study by Banerjees recently identified that picornaviral 3CD protein plays a crucial role as a master regulator during hijacking of the host cell phospholipid biosynthetic pathways; eventually resulting in proliferation of the membranes at the specific point [94]. This study demonstrated that 3CD viral protein alone is sufficient to induce PI4P, phosphatidylinositol-4,5-bisphosphate (PIP2) and phosphatidylcholine (PC) synthesis during picornaviral infections [94]. To this end, there is need to illustrate the mechanisms used by this viral protein to recruit an array of these cell membrane biogenesis lipids. To find out whether the formation of the replication organelle is conserved among the enteroviruses, Melia and colleagues studied the architecture of the replication organelles formed during encephalomyocarditis virus; a picornavirus in the genus *Cardiovirus* [95]. This study postulated that the endoplasmic reticulum might be the likely donor organelle for the formation of the replication organelle during EMCV infection [95]. The common belief that enteroviruses replication and evasion of the innate immune system signaling is aided by the formation of the membranous web was recently challenged by Melia and colleagues [96]. Using a known PI4PIIIβ inhibitor; BF738735 (identified in an earlier screen by van der Schaar and colleagues [97]), this study showed that a mutant coxsackievirus (CV-B3 3A-H57Y) was able to replicate within the Golgi apparatus in the absence of replication organelles [97].

To this end, the clear steps involved in the formation of the double membranous structures required for the formation of the enteroviruses replication organelles remain unresolved. There is need to dissect the exact mechanisms involved in the formation of replication complex; a mechanism without which the replication of the viral genomes becomes compromised. This might be an opening towards the development and or design of antivirals targeting this exact mechanism. For example, the mechanisms of the cell remodeling during RNA virus infection has been mined by a recent study by Nguyen and coworkers [98]. This study identified fatty acid synthase and ceramidase as potential inhibitory target against rhinoviruses [98], highlighting the possibility of targeting lipid transfer during the replication organelle formation for possible therapeutics.

### Host factors involved in enteroviral protein translation

Translation of viral proteins upon release into the cytoplasm is cap-independent thus human host proteins binds to the viral type 1 internal ribosome entry site (IRES) for efficient replication. Some nuclear factors relocate to the cytoplasm during enteroviral infections where they bind to the internal ribosome entry sites (IRES); acting as internal ribosome entry sites trans-acting factors (ITAFs) thereby recruiting ribosomes to the site for protein translation. RNA binding protein; heterogenous nuclear ribonucleoprotein (hnRNP)A1 is known to shuttle from nucleus to cytoplasm during enteroviral infections [99, 100]. Lin and colleagues demonstrated that this RNA binding protein(RBP) is an ITAF and binds to 5’UTR of EV-A71 and Sindbis virus during viral infection thus enhancing viral protein translation [101]. Tolbert and coworkers demonstrated that hnRNP A1 binds specifically to the stem loop II of the EV-A71 IRES [102]. A follow-up study by the same group demonstrated that hnRNP A1 induces conformational changes upon binding to the stem loop II of the EV-A71 IRES leading to the enhanced viral protein translation [103]. HnRNP A1 has also been linked to regulation of replication in other viruses such as hepatitis C virus [104], human cytomegalovirus where it interacts with immediate early gene 2 protein [105], dengue virus [106] and human papillomavirus type16 L1 [107] among other viruses.

Far upstream element binding protein 2 (FBP2) was described by Lin and colleagues to be an ITAF and a negative regulator of EV-A71 IRES dependent replication [108]. A follow-up study from the same group showed that EV-A71 induces proteasome, autophagy and caspase activity mediated cleavage of FBP2 into a positive regulator of viral protein synthesis [109]. FBP1; another nuclear protein was also demonstrated to translocate to the cytoplasm during EV-A71 infection where it binds to the viral IRES there by recruiting ribosomes to the sites for enhanced viral protein synthesis; thus, acting as a positive ITAF [110]. Studies by Zhang and coworkers described nuclear factor cellular factor 68-kDa Src-associated protein in mitosis (Sam68) as an EV-A71 positive ITAF; upon translocation into the cytoplasm [111].

Human host factors-viral protein studies identified nuclear factor; adenosine-uridine (AU)-rich element RNA binding factor 1 (AUF1) is targeted for cleavage by CV-B3 viral 3C protease upon translocation to the cytoplasm for enhanced stability of the IRES dependent viral RNA production [112], similar antiviral observations were made for poliovirus, coxsackievirus and human rhinovirus [113]. Rozovics and colleagues reported a 3CD dependent cleavage of AUF1 during poliovirus and rhinovirus infections enhances RNA replication [114]. Interestingly, the replication of another picornavirus; EMCV was not affected by messenger RNA decay protein: AUF1 as observed in other enteroviruses, suggesting a variance in the restriction mechanism of this nuclear factor [115]. Investigating the role of AUF1 in EV-A71 infections, Lin and colleagues showed that it relocates to the cytoplasm during infection where it binds to the viral IRES and restricts viral RNA production [116]. AUF1 is the only nuclear factor which has shown an effect on the replication of other picornaviruses; pointing to its possible global role during these viral infections, offering a possible target for in the development of antivirals against enteroviruses.

Other host factors described to be involved in picornaviral translational activity include; Misshapen NCK-related kinase (MINK) in EV-A71 [117], heterogenous nuclear ribonucleoprotein C [118], La autoantigen in hepatitis C cap-independent translation [104], polypyrimidine tract-binding protein (PTB) and poly(rC)-binding protein (PCBP) for IRES dependent translation of poliovirus [119], double stranded RNA binding protein 76 (DRBP76) acting as a negative IRES regulator for rhinovirus 2 [120, 121], as well as ploy(rC) binding protein 1 and 2 boosting poliovirus and rhinovirus IRES dependent translation [122].

The mode of action of enterovirus IRES is not fully understood as seems to be a myriad of host nuclear factors involved in the cap-independent viral replication. There is need for further research to help identify all the host factors involved in enteroviral IRES dependent RNA production. Identifying host factors that binds to the IRES during enterovirus cap-independent viral translation will be key in understanding the viral replication cycle.

### Programmed cell death during EV-A71 viral infection

Neuronal cell death as a result of enteroviral infections have been observed in some cases of HFMD [5, 123] and the mechanism linked to programmed cell death. For a long it has been a common belief that apoptosis and necrosis are the major players in programmed cell death (reviewed [124]). Other mechanisms including pyroptosis and necroptosis have been described to play a role in complementing apoptosis in restricting viral infections [125–128].

The process of caspace-1 induced pyroptosis was first described in *Salmonella enterica* serovar Typhimurium bacteria [129]; and has been elucidated to be used by other species of bacteria to escape inflammasome and stimulate cell death (reviewed [130]). Pyroptosis; inflammatory programmed cell death, has been linked to cell death during EV-A71 infections in neuronal cell lines [131]. AIM2 mediated inflammation had been linked to the pyroptosis during EV-A71 infections as it was up-regulated as well as AIM2 downstream stimulated genes such as CARD16, caspase-1 and IL-1β during viral infection in neuronal cell lines (SK-N-SH) [132]. Yogarajah and coworkers recently identified radical *S*-adenosylmethionine domain containing 2 (RSAD2) and Absent in melanoma 2 (AIM2) to be modulating EV-A71 and CV-A16 infections of the neuronal cells [133]. Consistent with previous findings from the same research group; the upregulation of AIM2 resulted in reduced viral replication [132]. The results from this study points to mechanisms involved in the neuronal complications observed in the fatal cases of EV-A71 infections which are not observed during CV-A16 infections. This observation is postulated to be as a result of differential stimulation of host factors during viral infections by the viral 5’non-tranlsated regions [133]. Involvement of pyroptosis during viral infection has been reported for other viruses including; encephalomyocarditis virus (EMCV) [134], rhinovirus [135] and adenoviruses [136].

### Enteroviruses induces cell cycle arrest for genome replication

Viruses are known to target various host cellular factors for effective and efficient replication. Several viruses have been shown to target human host cell cycle; arresting the cell division thereby avoiding competition from the dividing cells for their efficient genome replication. DNA viruses have been shown to have the ability of entering the cell cycle S phase and arresting cycle for viral replication; for example Simian Virus 40 [137], human papillomavirus 16 and 18 viral protein E6 interacts with p53 [138] as well as herpes simplex virus ability to block cell cycle is reviewed in details by Flemington and colleagues [139], have been shown to usurp the cell cycle for efficient viral replication process. Infectious bronchitis virus (IBV); a coronavirus was shown by Li and colleagues as well as Dove and coworkers to induce cell cycle arrest during the S and G(2)/M phases for improved viral replication [140, 141]. Influenza A virus replication has been shown to interact with cell division factors resulting to arrest of the cell cycle division at the G_0_ /G_1_phase [142]. Arrest of cell cycle at G_2_ phase by Human Immunodeficiency Virus-1 (HIV-1) viral protein R (Vpr) through blocking of p34cdc2/cyclin B complex stimulation [143, 144]. Coronaviruses; severe acute respiratory syndrome and mouse hepatitis virus (MHV) are able to capture cell cycle at G_0_/G_1_ phase for efficient genome replication [145–147].

Among enteroviruses, the cell cycle arrest has been reported for EV-A71, CV-A16, EV-D68 and recently for CV-A6 viruses. Targeting the cell cycle host factors helps the viruses to replicate within the cells with limited competition from actively dividing cells. Completely understanding the how viruses take advantage of the cellular processes/ proteins to establish efficient infection and genome replication is vital in the development of vaccines and antiviral therapy against these viruses.

Disruption of cell cycle division at S phase has been reported during EV-A71 infection thereby blocking the entry of the cells into G2/M phase through viral RNA dependent RNA polymerase 3D non-structural protein [148]. This study showed that EV-A71 mediates cell cycle through increasing transcription of cyclin E1, promoting proteasomal degradation of cyclin A2 and eventual phosphorylation of cyclin dependent kinase 2(CDK2) thus regulating expression of these key cyclin regulators [148]. The same study also showed that another picornavirus; coxsackievirus A16 infection as well mediates cell cycle division disruption at S phase [148]. Factors that control cell cycle and differentiation; Aurora B kinase (AURKB) and cyclin dependent kinase 6 (CDK6) were identified by Wu and colleagues as EV-A71 restriction factors [82].

EV-D68 mediates synchronization of cell division at the G_0_/G_1_ but not at S phase thus promoting viral replication while cell cycle arrest at G2/M phase inhibited viral replication [149]. This observation is contrary to CV-A16 and EV-A71 where cell arrest at S phase promoted viral replication. Remarkably, cell cycle disruption at G2/M phase inhibited viral replication for CV-A16, EV-A71 and EV-D68 viruses [148, 149]. Wang and colleagues demonstrated for the first time that CV-A6 disrupts cell division cycle at G0/G1 phase for viral replication through its non-structural protein RNA-dependent RNA polymerase 3D and 3C protease proteins [150]. Viruses depends on host cell proteins and processes for efficient genome replication. Exploiting the cell cycle process, a highly regulated process enables viruses to have unfettered access to the cell cycle factors for efficient viral replication.

Future work should look at the cell cycle stage where other enteroviruses disrupt cell division cycle. This will enable better antiviral therapy design and development targeting different viruses associated with HFMD as well as other forms of enteroviral infections.

### Role of autophagy in Enteroviral RNA replication and egress

The process of autophagy has been linked to the formation of the double membranous structures which acts a replication site for enteroviruses including poliovirus (PV). The formation of these membranous structures is dependent on the exploitation of the autophagy process by the enteroviruses (PV, CV-B, CV-B3 among other enteroviruses) where 3A and 2 BC viral proteins are involved [151–156]. Recent studies have linked autophagy regulators to the formation of the autophagosome/ the replication organelle during coxsackievirus B (CV-B); thus showing that enteroviruses not only target the autophagy process but also its regulators for efficient replication of their genomes [157, 158]. Wong and colleagues showed that coxsackievirus B3 (CV-B3) induces autophagosome formation without lysosome degradation of proteins [156], clearly highlighting the role of autophagosome in the formation of the replication organelles during enteroviral infections. Follow-up studies by Zhai and colleagues observed formation of autophagosomes both in CV-B3 infected fibroblasts and in Balb/c mice thus, linking autophagy to the pathogenesis of myocarditis infections [159]. The shedding of CV-B3 virus from infected cells was linked by Robinson and colleagues to the extracellular microvesicles with autophagosome markers. The role of the autophagomes in the release of CV-B3 virus from infected cells was later validated by Sin and coworkers [160]. The study by Sin and colleagues demonstrated ability of CV-B3 to egress from cells and infect other cells via a dynamin related protein 1(DRP1) initiated mitochondrial fragmentation; a process vital for the mitochondrial based autophagy elimination/mitophagy [161]. From this study, CV-B3 is believed to localize in the mitochondria where it initiates virus induced mitophagy and eventual escape from cells through autophagosome-bound-mitochondrion-virus complex [161]. The role of mitophagosome in the release of CV-B3 virus, explains possible alternative process used by picornaviruses to release from infected cells and infect other cells thus ensuring the infection cycle is sustained. The disruption of the mitochondrial dynamics through virus induced stimulation of DRIP1 to block virus induced apoptosis and eventual persistence of viral infection has also been observed in HCV [162]. This points to the fact that different single stranded RNA viruses may be using the same process to disrupt mitochondrial traffic and eventual apoptosis for viral replication maintenance of viral infection cycle.

Enterovirus A71(EV-A71) induced autophagy had been reported both in vivo and in vitro with EV-A71-VP1 and 2C proteins localizing with microtubule-associated protein 1 light chain 3(LC3) and mannose-6-phosphate receptor (MPR) resulting in the formation of the amphisome thereby increasing viral replication [163, 164]. EV-A71 2 BC non-structural protein was recently shown to trigger the formation of autolysosomes in human rhabdomyosarcoma cells thus enhancing EV-A71 replication [165]. This study also showed that the 2 BC protein interacts with *N*-ethylmaleimide-sensitive factor attachment receptor (SNARE) protein, syntaxin-17 (STX17), synaptosome associated protein 29 (SNAP29) and microtubule-associated protein 1 light chain 3B (LC3B) major players in the formation of the autolysosome [165]. The results of this study are consistent with earlier findings linking enterovirus 2 BC non-structural proteins to the exploitation of autophagy process to support enterovirus viral replication. Corona and colleagues showed that enterovirus D68(EV-D68) is able to disrupt autophagy processes downstream to promote viral replication and eventual egress from the cells thus promoting viral infection within the cells [166]. This phenomena linking viral proteins to interact with various regulators of autophagy processes for efficient viral replication and transmission has been reviewed [167, 168]. Another pending issue has been if the enteroviruses are able to replicate inside the acidic autophagosomes and how do they evade degradation and exit the cells intact. However, this has so far been linked to the enteroviruses’ ability to divert cargo traffic away from degradation [166, 169, 170]. CV-B3 3C protease has been illustrated to target cleavage of SNARE and PLEKHM1 proteins which are key in regulation of autophagosome fusion and eventually impairing establishment of SNARE complexes [170].

The role of autophagy regulators in enterovirus infections have also been studied. For example, a study by Delorme-Axford showed that an autophagy regulator; bactericidal/permeability-increasing protein (BPI) fold-containing family B, member 3 (BPIFB3) acts as a host limiting factor during coxsackievirus B virus infection [158]. This study reported that BPIFB3 may be playing a role in downregulating the key steps involved in the autophagy process proposed to be helping in the formation of the membranes needed for enteroviruses replication [158]. A study by Morosky and colleagues linked BPIFB6, another protein in the family of BPIFB to be a positive regulator of CV-B suggesting that BPIFB family of proteins may be having diverse effects in regulating viral infections [157]. A recent study by Delorme-Axford and coworkers identified exoribonuclease Xrn1 as a negative post-transcriptional regulator of autophagy [171]. The same study also showed that Xrn1 maintains the process of autophagy at basal levels thus limiting replication of poliovirus and coxsackievirus B [171].

A recent study by Velazquez and colleagues demonstrated that poliovirus can generate autophagosomes through a downstream of ULK1 signaling pathway; cleaving the cargo traffickers which may negatively interfere with cargo loading [172]. This points out to the ability of the picornaviruses to fine-tune the interaction with the autophagy machinery for effective survival within the cells. Targeting of autophagy key players and auxiliary factors have been reported for number of picornaviruses. CV-B3 through its viral 2Aprotease was has been shown to cleave sequestosome 1/p62 (SQSTM1/p62) [173]; a known intermediary of selective autophagy degradation of ubiquitinated proteins [174–176]. This study further showed that cleavage of SQSTM1 resulted in the impairment of NF-kB signaling and eventual disruption of the selective autophagy in infected cells; emerging as an pro-viral strategy to establish an efficient infection during CV-B3 infection [173]. A subsequent study by Mohamud and colleagues demonstrated that SQSTM1 and another host factor calcium binding and coiled-coil domain-containing protein 2/nuclear dot 10 protein 52 (CALCOCO2) regulate CV-B3 virus infection by targeting autophagy receptors; via their interaction with viral protein 1 [177]. This study also showed that CALCOCO2 targets mitochondrial antiviral signaling protein for degradation thereby blocking the establishment of antiviral state within the infected cells for efficient establishment of CV-B3 infection [177]. Different strategies used by viruses to trigger and hijack the process of autophagy have been recently reviewed in details by Zhang and coworkers [178].

Autophagy is key in controlling various cellular processes including enhancing innate immune signaling during viral infections through a process known as virophagy. The ability of virus infected mitophagosomes to be released out of the infected cells provides an important mechanism of virus egress from the infected cells. Enteroviruses have been shown to have the ability of interacting with cellular autophagic process that is conventionally known to degrade the mitochondrial traffic upon fusion with the lysosomes. Enteroviruses has evolved ways to evade this process through degradation of various autophagy initiating factors as well as its regulators. This host cellular process has been linked to the non-lytic exit of various enterovirus infections including Poliovirus, Echovirus 7, EVA71 and CV-B3 viruses. However, blocking initiation of mitophagy as a way of controlling viral infections may not be feasible given that observations from different studies have shown only disruption of extracellular micro-vesicles (EMV) release and not the replication ability of CV-B3 virus. Thus, this process does not provide an ideal antiviral target. An overview of the human host cell/process: NPEV viral protein interactions are highlighted in Table 2 below.

**Table 2:** host factors involved in NPEV infection cycle

Human host factor: Viral Protein interaction	Role during viral infection cycle	Reference
EV-A71 3C^pro^ Cleaves Ubc6e at Q219G, Q260S and Q273G	Inhibits ERAD pathway to promote viral replication	[84]
EV-A71 2A^pro^ Inhibits synthesis of Herp and VIMP ER proteins at translational level		
EV-A71 3A facilitates interaction with ACBD3 and PI4PIIIβ at replication site for formation of replication complexes	Formation of membranous structures for viral replication	[93, 94]
Picornaviral 3CD protein induces PI4P and PIP2 and phosphatidylcholine synthesis during picornaviral infections		
hnRNP A1 relocates to cytoplasm from nucleus and binds to the stem loop II of the EV-A71 IRES	Viral protein translation: Enhanced IRES dependent viral RNA production	[99–103, 109, 110]
EV-A71 induces proteasome, autophagy and caspase activity cleavage of FBP2 into a positive ITAF		
Sam68 translocates into the cytoplasm and binds to viral IRES		[111, 112, 114, 117]
CV-B3, rhinovirus viral 3C protease cleaves AUF1 upon translocation to the cytoplasm		
MINK binds to IRES acting as an ITAF		
EV-A71 viral 3D RNA dependent RNA polymerase disrupts cell cycle division at S phase thus blocking entry into G2/M phase	Cell cycle arrest for efficient replication by accessing the host factors cell division machinery.	[148–150]
EV-D68 mediates synchronization of cell division at G0/G1		
CV-A6 viral protein 3D and 3C disrupts cell division cycle at G0/G1		
PV, CV-B, CV-B3 virus induced autophagy through 3A and 2 BC viral proteins	Formation of replication complexes for viral replication.	[151–156, 159]
CV-B3 induces autophagosome formation without lysosome degradation in fibroblasts and BALB/C mice		
CV-B3 induces DRP1 initiated mitochondrial fragmentation	Virus egress through the autophagosome-bound-mitochondrion-virus complex	[161]
EV-A71-VP1 and 2C proteins induce autophagy through localization with LC3 and MPR	Enhanced EV-A71 replication through formation of amphisome	[163, 164]
EV-A71 2 BC protein interacts with SNARE, STX17, SNAP29 and LC3B proteins leading to formation of autolysosome in RD cells	Enhanced viral replication	[165]
EV-D68 can disrupt autophagy process downstream	Promotes viral replication and egress from infected cells; promoting viral infection within the cells	[166]
CV-B3 viral protein 3C targets cleavage of SNARE and PLEKHM1 proteins	Impairs establishment of SNARE complexes thus providing conducive environment for viral replication	[170]
CV-B3 viral 2A protease cleaves SQSTM1/p62 a known intermediary of selective autophagy degradation of ubiquitinated proteins	Impairs NF-kB signaling and disrupts selective autophagy in infected cells to establish an efficient viral replication/infection	[173]
CV-B3 interacts with CALCOCO2 and SQSTM1	Targets autophagy receptors; targets mitochondrial antiviral signaling protein for degradation thus blocking establishment of antiviral state in the infected cells	[177]

### Advances in enterovirus antiviral drug developments

Much has not been achieved in the development of antivirals against NPEV infections. The major challenge for development of the antivirals has always been the mutations on the viral genomes. Several compounds have been tested for possible use as antivirals against enteroviruses as shown in Table 3 below but no much success has been achieved. Most of the drug screening have been done in vitro with little success in vivo and in clinical trials. Screening FDA approved drugs and repurposing of existing drugs based on known viral-human protein interactions are some of the strategies that has been adopted by scientists to identify antivirals against NPEVs. For example, Li and colleagues evaluated the effects of ribavirin a known antiviral against other RNA viruses on EV-A71 for possible repurposing of the drug [179]. Their study showed decreased EV-A71 virus yield in vitro and reduced disease status, death and adverse effects associated with its infection in vivo; highlighting the possible role as an antiviral compound against EV-A71 [179]. Plant metabolites have also been targeted as possible antiviral compounds against enteroviruses. For example, Quercetin; a well distributed plant flavonoid has been shown recently to inhibit EV-A71 infection by inhibiting virus attachment, adsorption and by targeting viral 3C protease [180].

The antiviral efficacy of pyrazolo[3,4-d]pyrimidines have also been evaluated against enteroviruses; CV-B3 and EV-A71 virus infections where they inhibited their infections but the exact mechanism was not established [181]. More recently, andrographolide has been reported to suppress EV-D68 replication targeting the viral maturation within the acidified endosomes [182]. World Health organization (WHO) recommended combination therapy has also been evaluated for possible antiviral development against enteroviruses [183]. Screening of FDA approved drugs recognized pirlindole as a strong inhibitor of CV-B3 [184].

Natural products have recently gained much interest in drug development studies. Of these; plant secondary metabolites; flavonoids have been of interest in drug therapy screens against viral infections given that they are freely available and form better part of human dietary. Screening of plant metabolites for possible use as antiviral therapy has been reported as reviewed by Zakaryan and colleagues [185] and their biological activity as well as chemistry have also been extensively reviewed [186]. Some flavonoids with antiviral abilities in vitro against viral infections include; isoquercitrin against Zika virus infections [187], chikungunya infections [188], apigenin antiviral effects on a number of viruses such as African swine fever virus (ASFV), hepatitis C virus [189, 190]. Apigenin have also shown antiviral activity against EV-A71 virus by inhibiting viral IRES dependent translation [191–193]. A recent screen of flavonoid library identified ST077124 and ST024734 as lead antiviral compounds against EV-A71, CV-A6 and CV-A16 enteroviruses [194]. All these concerted efforts towards identifying antivirals against enteroviruses and other viral infections needs a follow-up and validation in animal models. The good news is that most of the already identified compounds have shown no cytotoxicity in cells; thus, may not have toxic effects in animal models. The efficacy of most of the identified compounds have only been elucidated in vitro thus there is need for further studies to identify their effects in vitro.

**Table 3:** Non-Poliovirus Inhibitors

Drug compound	Virus	Mode of action	Reference
Andrographolide	EV-D68	Suppresses maturation of the virus within the acidic endosomes.	[182]
DTriP	EV-A71	Blocks viral replication by targeting the RNA dependent RNA polymerase.	[195]
Ribavirin	EV-A71	General reduction of the viral yield	[179]
Quercetin	EV-A71	Inhibits virus attachment, adsorption and the viral 3C protease	[180]
Rupintrivir	EV-A71, CV-A16, HRV	Binds and inhibits viral 3C protease activity	[196–198]
Fluoxetine	CV-B3	Reduces synthesis of viral RNA and protein	[199]
Dibucaine	CV-B3, EV-D68,	RNA replication stage; may be targeting CV-B3 viral 2C protease	[184]
Formoterol	CV-B3, EV-D68, EV-A71, RV-A2, RV-B14	Likely inhibit RV-14 by reducing ICAM-1 levels or acidic endosomes.	[184, 200]
Pirlindole	EV-D68 and CV-B3	RNA replication stage; may be targeting CV-B3 viral 2C protease	
Budesonide	RV-14		[200]
Zuclopenthixol	EV-D68 and CV-B3	RNA replication stage; may be targeting CV-B3 viral 2C protease	[184]
Apigenin	EV-A71	Targeting viral IRES thereby inhibiting viral translation process	[191–193]

Little success has been achieved in terms of antiviral therapy against enteroviruses. Given that drug discovery process is an expensive and time-consuming venture, most researchers have relied on the FDA approved drugs or drugs that are already in use for possible re-purposing. Not much success on drug therapy has been recorded in viral infections due to the high mutation rates observed during viral replication. Combination therapy of the drugs with different mode of actions targeting different stages of viral infections would be an alternative in targeting different stages of the enteroviral infection cycle. This will only be achieved with a complete map out of the human host factors hijacked by these viruses during infections. Thus, there is need for continued elucidation of molecular mechanisms of the already postulated viral targets as well as identifying other underlying factors and process. Vaccines have shown much success against viral infections and the success story of vaccination against poliovirus infection in the world which is a picornavirus; points for the need of continued studies towards identifying vaccine candidates against the enteroviral infections. With outbreaks of enteroviruses being recorded in different parts of the world, if not checked they might have a potential threat to the global health; just soon after near- eradication of poliovirus infection.

## Conclusion and future perspectives

The emergence of outbreaks of enteroviral infections in different parts of the world point to the need of mapping all the host factors involved in the infection paradigm. Given that viruses need host factors in every step of their infection from attachment, entry, replication, virion assembly and eventual entry, there is need to elucidate all the host factors involved for an improved understanding of the molecular dynamics of enteroviral infections. This will be a big boost towards the long overdue antiviral and vaccine development against these epidemiologically important viruses. There is much to be elucidated on the formation of NPEV replication complex formation as the existing mechanisms do not wholly explain the processes and steps involved in this important process during viral replication. The nuclear host factors involved in the enteroviral replication also needs to be fully described as this is a vital step in maintaining viral replication and eventual life cycle. Viral entry studies need to be carried out as the known receptors and viral entry requirements do not fully explain the myriad of disease features observed during viral infections. The role of cellular processes such as autophagy, apoptosis, necroptosis, pyroptosis as well as post-translational modifications in enteroviral infections also needs to be fully elucidated. This will be specifically important in explaining the little-known stages of viral infections such as non-lytic egress for continuous viral cycle within the host.

The paucity of information on the infection dynamics of these viruses calls for concerted efforts to elucidate the viral-human cell interactions. There is still a lot to be investigated to fill the gaps that exist on the life cycle of non-polio enteroviruses. With new cases emerging in different parts of the world, it is just a matter of time before we have a global outbreak of non-poliovirus enteroviral infections in different parts of the world. There is also an urgent need for further studies especially in the field of vaccine developments as well as antiviral therapy against enteroviruses.

## Data Availability

Not applicable.
